# Development and validation of the Decisional Balance Scale for Physical Activity in Female Survivors of Violence (DBSPA-FSV)

**DOI:** 10.3389/fpubh.2025.1638237

**Published:** 2025-10-28

**Authors:** Camille Favola, Fabienne d'Arripe-Longueville, Lou-Anne Piroird, Meggy Hayotte, Stéphanie Mériaux-Scoffier

**Affiliations:** ^1^Université Côte-d'Azur, LAMHESS (UPR 6312), Nice, France; ^2^Institut Universitaire de France, Nice, France; ^3^Centre de Recherche de l'Institut Universitaire en Santé Mentale de Montréal, Université du Québec à Trois-Rivières, Montréal, QC, Canada

**Keywords:** psychometric validation, transtheoretical model, behavior change, facilitators and barriers, self-determined motivation, physical activity engagement, women's health

## Abstract

**Introduction:**

Due to the trauma they have experienced, women who are survivors of violence struggle to engage in regular physical activity despite its numerous benefits. Identifying the factors that facilitate or hinder engagement in physical activity within this population is therefore essential. However, no valid tool currently exists specifically for this purpose. This study, based on the concept of decisional balance drawn from the transtheoretical model of behavior change, aimed to develop and validate the Decisional Balance Scale for Physical Activity in Female Survivors of Violence (DBSPA-FSV).

**Methods:**

Three hundred one volunteers participated in three complementary steps which followed established validation procedures. In step 1, a preliminary version of the items was developed based on the existing literature. In step 2, the dimensionality and convergent validity of the scale were examined. In step 3, the reliability of the scale was tested.

**Results:**

In step 1, a preliminary version of 32 items was developed. The scale was refined to 22 items, grouped into two factors (facilitators and barriers) and six sub-dimensions (physical, psychological, and socio-environmental). In step 2, bi-factor confirmatory models with a global construct and six or two correlated factors demonstrated satisfactory fit indexes. Convergent validity was confirmed by significant correlations between DBSPA- FSV constructs and the concept of self-determined motivation in the expected directions. In step 3, the internal and test-retest reliability of the scale were confirmed.

**Discussion:**

The DBSPA-FSV scale exhibits satisfactory psychometric properties and will contribute to research on the engagement in physical activity of women survivors of violence.

## 1 Introduction

Violence against women exists along a continuum, spanning psychological abuse, physical aggression, and sexual violence (1). It represents a critical global public health concern. According to the United Nations, violence against women includes all forms of gender-based violence that cause physical, sexual, or psychological harm or suffering, encompassing threats, coercion, or arbitrary deprivation of liberty, whether occurring in public or private settings (2). This is the definition of violence used in this study. Globally, ~736 million women—nearly one in three—have experienced physical and/or sexual violence by an intimate partner, non-partner, or both, at least once in their lifetime (3). Despite this staggering prevalence, underreporting remains widespread. A 2021 World Health Organization report revealed that fewer than 40% of women who experience violence seek any form of support, and of those, <10% contact the police (4). Silence among survivors is often driven by fear, stigma, and a deep-seated mistrust in judicial systems (4). In some national studies, fewer than one in 10 rape complaints lead to a conviction. These statistics highlight a disturbing reality: official reports capture only a fraction of the true extent of gender-based violence (5).

Violence can have profound and sometimes delayed effects on physical and mental health, particularly in the form of psychological trauma. Childhood violence, in particular, has been shown to lead to long-term consequences that may persist regardless of any violence experienced in adulthood (6). The more physical violence individuals endure, the more their quality of life deteriorates, affecting both their health and social relationships (7). These effects extend beyond childhood, as survivors of sexual violence frequently report physical symptoms such as migraines, fatigue, eating disorders, and various forms of chronic pain (7). The long-term psychological and physical consequences of violence (particularly when experienced in childhood) highlight the need for holistic responses. Non-pharmacological approaches such as physical activity (PA) have emerged as promising avenues for support and recovery. Several studies have highlighted the positive impact of PA on the physical and mental health of survivors. A meta-analysis showed that PA programs significantly reduce post-traumatic stress disorder (PTSD) symptoms compared to control groups (8). Other authors have found that PA not only alleviates PTSD symptoms but is especially effective for individuals resistant to conventional treatments, reducing anxiety, depression, sleep disorders, and cardiovascular problems often linked to PTSD (9). Moro (10) demonstrated that PA reduces the risk factors for developing metabolic diseases, which are exacerbated by a sedentary lifestyle, and advocated for its use as a first-line treatment. Participation in PA programs can reduce the risk of chronic diseases, lower mortality, and improve mental health, especially among individuals with PTSD, as confirmed by a systematic review (11). The World Health Organization has also emphasized the broad health consequences of violence, including chronic pain and general health deterioration. In this context, tailored physical activity could offer a meaningful avenue for improving both physical and mental wellbeing (12). More specifically, high-intensity exercises appeared to be more effective in reducing PTSD symptoms than moderate-intensity exercises (13). Furthermore, Basile et al. (14) confirmed that PA effectively combats mild to moderate depression, alleviates major depression symptoms, and lowers mortality rates. Despite these benefits, women who have experienced violence often face significant barriers to engaging in PA, leading to increased sedentary behaviors (15, 16). Understanding and addressing these barriers is crucial to promoting PA engagement in this population.

Several studies have highlighted the positive impact of physical activity (PA) on the physical health of survivors (17). Psychological trauma—defined as “exposure to actual or threatened death, serious injury, or sexual violence” (DSM-5-TR) (18) can lead to post-traumatic stress disorder (PTSD), one of the most recognized and debilitating manifestations of trauma. PTSD symptoms are grouped into five main clusters: intrusive memories and flashbacks, avoidance behaviors, negative alterations in cognition and mood, heightened arousal and reactivity (including sleep disturbances and irritability), and dissociative symptoms such as depersonalization or derealization. Given the complexity and persistence of these symptoms, PTSD represents a major public health concern, affecting ~5%−12% of the general population, with significantly higher prevalence among survivors of violence (19). In this context, physical activity has emerged as a promising complementary intervention. The beneficial effects of physical activity (PA) on mental health are supported by several mechanisms. Neurobiologically, PA helps reduce stress reactivity, improve mood regulation, and enhance sleep quality (8). Psychosocially, it contributes to strengthening perceived self-efficacy, reducing feelings of isolation, and providing a constructive outlet for stress management (17). A meta-analysis has shown that PA programs significantly reduce PTSD symptoms compared to control groups (8). Similarly, other studies have found that PA is particularly effective for individuals resistant to conventional treatments. Research has demonstrated that PA contributes to reducing anxiety, depression, sleep disturbances, and cardiovascular issues often associated with PTSD (9). Physical activity (PA) reduces risk factors for metabolic diseases linked to sedentary lifestyles and is recognized as a first-line intervention and potential alternative to medication (10). Evidence also shows that PA lowers mortality rates, decreases the risk of chronic conditions, and enhances mental health (11). The World Health Organization emphasizes the wide-ranging health consequences of violence, including chronic pain and general health decline, highlighting the value of tailored PA for improving both physical and psychological outcomes. High-intensity exercise appears particularly effective in reducing PTSD symptoms, and PA more broadly has been shown to alleviate depression and lower mortality risk (14). Yet, women who have experienced violence often face significant barriers to engaging in PA, resulting in greater sedentary behavior (15, 16). Addressing these barriers is crucial to promoting recovery in this vulnerable population.

Barriers to engaging in PA can be psychological, social, environmental, or physical. Among these, psychological barriers play a significant role in limiting PA participation of women survivors of violence. These include: (a) fear, often stemming from past experiences (19); (b) psychological trauma, which can negatively affect their ability to engage in new or demanding activities (20); (c) loss of self-confidence and social withdrawal, which is an obstacle to their participation in group activities (19). Some survivors also experience physiological arousal during exercise—such as increased heart rate or breathing—as reminiscent of traumatic experiences, which they perceive as threatening or overwhelming. This form of hyperawareness of bodily sensations can lead to avoidance of physical activity altogether, particularly among survivors of sexual violence (21). Social and physical environments can also be seen as major obstacles: social isolation, which reduces opportunities to participate in structured or group activities, and environments perceived as a threat to their physical and emotional wellbeing (15). Finally, some women perceive PA as a threat to their physical integrity and consider it dangerous, which reinforces their reluctance to participate (15). To overcome these obstacles, it is essential to put in place supportive conditions or strategies tailored to the needs of women who have experienced violence, in order to encourage their engagement in physical activity. These women must feel comfortable in their social environment and establish meaningful connections with other participants (20). Intervention teams should foster a culture of trust, respect, and confidentiality to ensure a safe space for participants (17). In this perspective, recent studies highlight the importance of encouraging these women to exercise and improve their overall wellbeing by offering interventions that take into account the trauma they have experienced (17). Additionally, studies point out that it is essential for them to experience the benefits of exercise, both physically and psychologically, to maintain their engagement in PA (22). Specific PA programs for women survivors of violence are therefore highly justified. However, no existing study has provided a theoretical framework to explain behavior change or offered a validated tool to quantitatively measure PA engagement within this specific population (16). It is therefore essential to develop decision balance questionnaires based on strong theoretical foundations and tailored to the unique needs of this population to measure what encourages or hinders their participation in PA. Decision balance questionnaires, which assess the perceived pros and cons of engaging in a behavior such as physical activity, provide insight into the motivational processes underlying behavior change (23).

One particularly useful framework for explaining health behavior change, including physical activity (PA), is the Transtheoretical Model of Behavior Change (24, 25). This model is especially relevant in this context as it conceptualizes the change not as a single event but as a dynamic, staged process. This perspective is particularly appropriate for survivors of violence, whose engagement in PA may be shaped by fluctuating psychological readiness as well as unique barriers and facilitators. Unlike other models that focus on static determinants of behavior, the model highlights individual readiness to change and supports the tailoring of interventions to specific needs. The model comprises five stages—precontemplation, contemplation, preparation, action, and maintenance—with progression occurring in a non-linear manner depending on personal experiences. Two central mechanisms underpinning the model are self-efficacy, which reflects an individual's confidence in their capacity to achieve change, and decisional balance, which involves weighing the perceived benefits and drawbacks of adopting a behavior.

The concept of decisional balance, introduced in 1977 by Janis and Mann (23) and later integrated into the Transtheoretical Model of Behavior Change (24, 25), refers to an individual's cognitive evaluation of the perceived benefits (pros) and perceived costs (cons) of adopting a specific behavior. This framework helps to understand the motivational processes that influence behavior change and resistance. In the context of physical activity (PA), decisional balance provides a structured way to assess the facilitators and barriers that individuals weigh when deciding whether to engage in PA. In 1992, Marcus et al. (26) developed an English-language version of the decisional balance scale for physical activity (PA), which evaluates these facilitators and barriers. Eeckhout et al. (27) later translated this scale into French for use in the general population. However this version does not account for the psychological dimensions specific to women who have experienced violence (e.g., no emphasis on trust, security and confidentiality was needed) (27). Other decisional balance tools have been developed for specific contexts, such as the workplace (28) or for populations with particular health conditions like cystic fibrosis (29) or arthritis (30). However, these tools were designed for specific groups with different facilitators and barriers with respect to PA than those perceived by women survivors of violence. For example, the decisional balance developed (29) in the context of cystic fibrosis includes items related to this genetic disease that affects the respiratory system (e.g., “good physical condition supports transplant success”). Such items are not applicable to our population. As a result, existing decisional balance tools are either too generic and do not take into account the unique needs of women survivors of violence, or, on the contrary, are too specific and therefore not applicable in the specific context of violence. In short, no tool is currently available to measure the facilitators and barriers for engaging in PA perceived by women survivor of violence, considering the psychological and physical trauma they have experienced.

The aim of this study was to develop and validate a Decisional Balance Scale for Physical Activity in Female Survivors of Violence (DBSPA-FSV) in a French sample. Three complementary steps were followed in accordance with recommendations for scale development and validation (31, 32). The items were developed by consensus by a group of experts, were based on the literature to ensure the validity of the scale content, and were subjected to additional analyses to check clarity (Step 1). Dimensionality and convergent validity of the DBSPA-FSV were examined through confirmatory factor analyses (CFA) and Pearson's correlations (Step 2). The convergent validity will be examined by investigating relationship between DBSPA-FSV with self-determination. Previous research indicates that individuals in early stages of readiness tend to be less self-determined in their physical activity behavior than those in more advanced stages (33). In the specific context of survivors of violence, a correlation between perceived barriers and lower motivation has also been reported (17). Specifically, we hypothesized that higher perceived facilitators (vs. barriers) will be associated with more self-determined forms of motivation. Finally, reliability of the scale was examined by measuring its internal consistency and its consistency across time (Step 3). For all steps, the protocol was approved by the local ethics committee for non-interventional research (authorization no. 2023-104) of University XXX. All participants were adults and individuals under 18 were excluded. Informed consent was obtained online via an information page and a checkbox before accessing the questionnaire. The questionnaires were administered online using LimeSurvey software version 3.17.3+ (LimeSurvey, CE), which guaranteed free participation, anonymity and confidentiality of the answers, and removed any possibility of missing data. No incentive for participation was provided. Statistical analyses were carried out using IBM SPSS and AMOS version 27 software (IBM Corp.). Finally, the study was open to all women, and no mention of violence was made in the recruitment materials. Whether women had experienced violence or not was assessed through self-report as part of the questionnaire, and all participants—both survivors and non-exposed women—were included in the analyses. This strategy preserved participant anonymity, avoided direct solicitation, and ensured a non-discriminatory recruitment process.

## 2 Step 1: development of a preliminary version of the decisional balance scale for physical activity in female survivors of violence (DBSPA-FSV scale)

The aim of this step of the study was to develop a preliminary version of the scale, by defining items that reflect the barriers and facilitators for engaging in PA specific to female survivors of violence based on the existing literature.

### 2.1 Method

#### 2.1.1 Development of the DBSPA-FSV scale

A committee of seven experts was set up, including: (a) four researchers and a master's degree student specialized in the fields of sport science, health psychology, and sport psychology, and (b) two representatives of associations working notably with survivors of violence. This committee developed items based on existing scales of decisional balance (28–30), on the studies by Pebole et al. (7, 15, 17) on the PA participation of women survivors of violence, and on a qualitative study of the barriers and facilitators for engaging in PA reported by women survivors of violence (22). Physical, psychological, social and environmental barriers and facilitators were identified. The committee created items for the following six subscales: (a) physical barriers, (b) psychological barriers, (c) socio-environmental barriers, (d) physical facilitators, (e) psychological facilitators, and (f) socio-environmental facilitators. These subscales could be grouped into two general subscales of barriers or facilitators. In line with the usual recommendations to have at least three items per factor to avoid estimation problems (29), the committee initially chose to develop four items per dimension. Item wording guidelines (34) and the principle of over-inclusiveness (31) were closely followed. Initially, the scale included 32 items (16 for facilitators and 16 for barriers). After several meetings, the experts' committee decided to remove eleven items (five facilitators and six barriers) which were redundant, lacked clarity or were ambiguous. At the end of this item development process, a 22-item scale was obtained, which included 11 facilitators and 11 barriers.

#### 2.1.2 Participants and procedure

Two analyses were conducted to assess the clarity of the scale. In the first step, a sample of 20 women aged 18–72 years (M_age_ = 32.05; SD = 15.16) from the south of France, with educational levels ranging from middle school diploma to PhD, rated the clarity of the 22-item DBSPA-FSV using a six-point Likert scale (one = not clear at all to six = completely clear). Participants were also invited to suggest alternative wording for any items they found unclear. Items with an average rating below 4.50 were revised accordingly. In the second step, a separate sample of 25 women (M_age_ = 38.96; SD = 17.87) evaluated the clarity of the revised version of the 22-item DBSPA-FSV (see Table 1). The two subsamples were recruited independently: the first group through convenience sampling within the researchers' networks, without criteria related to exposure to violence to ensure anonymity and avoid distress; and the second group from a larger pool of participants in the main survey. Both subsamples were independent and did not overlap with the main sample described in Section 3.1.1.

**Table 1:** The 22-item version of the decisional balance scale for physical activity in female survivors of violence (DBSPA-FSV).

**Dimensions**	**French version**	**English version**
Facilitators	Je pratique de l'activité physique parce que…	I'm physically active because…
**Physical facilitators (PHYF)**		
PHYF1	… cela me permet de repousser mes limites corporelles	… it allows me to push my physical limits
PHYF2	… je me sens gagner en énergie et en vitalité	… It helps me feel more energetic and lively
PHYF3	… je me sens plus forte physiquement	… I feel physically stronger
**Psychological facilitators (PSYF)**		
PSYF1	… j'ai repris confiance en moi	… It has helped me regain self-confidence
PSYF2	… cela me fait me sentir plus efficace dans mon travail	… it makes me feel more efficient at work
PSYF3	… cela me permet de libérer des émotions négatives	… it allows me to release negative emotions
PSYF4	… cela me permet d'évacuer les tensions	… it allows me to release tension
**Socio-environmental facilitators (SEF)**		
SEF1	… je me sens en sécurité sur mon lieu de pratique	… I feel safe where I exercise
SEF2	… je me rend sur mon lieu de pratique avec une personne de confiance	… I go to the place where I exercise with someone I trust
SEF3	… j'ai quelqu'un (un.e partenaire, un.e ami.e, autre) qui m'incite/me motive à pratiquer	… I have someone (a partner, a friend, other) who encourages/motivates me to exercise
SEF4	… j'ai un cadre de vie rassurant	… I live in a reassuring environment
Barriers	Je ne pratique pas d'activité physique parce que…	I'm not physically active because…
**Physical barriers (PHYB)**		
PHYB1	… j'ai des douleurs	… I'm suffering from pain
PHYB2	… je souffre de pathologies	… I'm suffering from a medical condition
PHYB3	… j'ai peur de me blesser	… I'm afraid of hurting myself
**Psychological barriers (PSYB)**		
PSYB1	… je ne me sens pas capable	… I don't feel up to it
PSYB2	… j'ai peur d'exposer mon corps	… I'm afraid of exposing my body
PSYB3	… je me sens fatiguée	… I feel tired
PSYB4	… je serais mal à l'aise	… I'd be uncomfortable
**Socio-environmental barriers (SEB)**		
SEB1	… je n'ai personne avec qui pratiquer/je n'ai pas d'ami. e sur mon lieu de pratique	… I don't have anyone to exercise with/I don't have any friends where I exercise
SEB2	… je n'ai pas le temps	… I don't have the time
SEB3	… je ne trouve pas de structure adaptée	… I can't find a suitable facility
SEB4	… je n'ai personne pour m'accompagner	… I don't have anyone to go with me

Each item is rated on a six-point Likert scale ranging from one (strongly disagree) to six (strongly agree). Score calculation: facilitators score (SF) = (PHYF1 + PHYF2 + PHYF3 + PSYF1 + PSYF2 + PSYF3 + PSYF4 + SEF1 + SEF2 + SEF3 + SEF4)/11; barriers score (BS) = (PHYB1 + PHYB2 + PHYB3 + PSYB1 + PSYB2 + PSYB3 + PSYB4 + SEB1 + SEB2 + SEB3 + SEB4)/11; decisional balance score (DBS) = FS—BS; interpretation of the DB score: DB > 0.5: facilitators outweigh barriers; BD comprised between −0.5 and 0.5: perfect balance between facilitators and barriers; BD < −0.5: barriers outweigh facilitators.

### 2.2 Results and discussion

An initial clarity analysis revealed rather satisfactory scores, with all items scoring at least 4.15/6 (M = 5.32; SD = 0.39). In response to the participants' recommendations, the items rated below 4.5 were rephrased as follows: (a) “I go to the place where I exercise accompanied” was replaced by “I go to the place where I exercise with someone I trust”; (b) “those close to me support me in my practice” were reworded as follows: “I have someone (a partner, a friend, other) who encourages/motivates me to exercise”; and (c) “I've changed my living environment” was replaced by “I live in a reassuring environment.”

The second clarity analysis resulted in a score of at least 4.48/6 for all items (M = 5.41, SD = 0.27). Therefore, the clarity of all items was considered satisfactory.

## 3 Step 2: dimensionality and convergent validity of the DBSPA-FSV

The second step of the study was to analyze the dimensionality of the DBSPA-FSV using a confirmatory factor analysis (CFA), and to examine its convergent validity by investigating its correlations with theoretically related constructs such as the different types of motivation for engaging in PA (35).

### 3.1 Method

#### 3.1.1 Participants and procedure

A total sample of 301 women (M_age_ = 19.76; SD = 5.80) survivors of violence (*n* = 136) and women not exposed to violence (*n* = 165), took part in the study. This sample was not limited to students but included participants with varied socio-demographic and educational profiles. All participants were included in the analyses. As a reminder, violence in this study is defined as any behavior along the continuum of violence, including psychological abuse, physical aggression, and sexual violence (36). Participants were recruited through social media and within university settings, via online posts and posters displayed in student-accessible areas. Additionally, brief presentations were made in large lecture halls to inform students about the study and reach a broader audience. Participants were asked to provide general information (e.g., demographic data), to complete the DBSPA-FSV scale, and take a survey that assesses the different types of motivation for engaging in PA. Participants' characteristics are presented in Table 2.

**Table 2:** Characteristics of the participants involved in checking the dimensionality and the convergent validity of the DBSPA-FSV scale (*N* = 301).

**Categories**	***N***	**Mean (SD)**
Age (year)	301	19.76 (5.80)
Height (m)	301	1.65 (0.06)
Weight (kg)	301	59.00 (9.72)
BMI (kg/m^2^)	301	21.60 (3.20)
**Years of education**		
< 12	4	
12	271	
15	17	
≥17	9	
**Exposed to violence**		
No	165	
Yes	136	
Number of years since violence occurred		3.04 (4.79)

Years of education: < 12: French junior High School Diploma, Professional Aptitude Certificate, Professional Studies Certificate; 12: High School Diploma; 15: Bachelor's degree; ≥17: Master's degree or PhD.

#### 3.1.2 Measures

##### 3.1.2.1 Demographic data

Information about gender, age, height, weight, highest degree and marital status were gathered. In addition, the presence or absence of violence experienced was measured (“Have you experienced violence in your life?”) after a description of the continuum of violence. In the event of violence, respondents were asked to provide the number of years since violence occurred.

##### 3.1.2.2 DBSPA-FSV scale

The 22-item scale developed in the previous step was used. Each item was rated on a six-point Likert scale (strongly disagree: one, to strongly agree: six).

##### 3.1.2.3 Motivation for physical activity (EMAPS)

Motivation for engaging in PA was assessed using the scale of motivation toward PA in a health context (37). This questionnaire includes 18 items rated on a Likert scale ranging from 1 to 7 (does not apply to me at all: 1, to perfectly applies to me: 7). The 18 items are classified into six types of motivation along a continuum (i.e., amotivation: lack of intention to act; external regulation: behavior driven by external rewards or pressures; introjected regulation: behavior motivated by internal pressures such as guilt; identified regulation: behavior recognized as personally important; integrated regulation: behavior fully aligned with one's values, all representing forms of extrinsic motivation; and intrinsic motivation: behavior undertaken for inherent enjoyment and interest). These can be grouped into self-determined forms (identified, integrated, and intrinsic) and non-self-determined forms (external, introjected, and amotivation). This classification makes it possible to distinguish motivations reflecting autonomous engagement in PA from those driven by external or internal pressures. Cronbach's alphas were above 0.79 and therefore considered fairly high (38).

#### 3.1.3 Data analysis

##### 3.1.3.1 Confirmatory factor analysis

As the development of the DBSPA-FSV scale was based on the literature, the number of factors were defined *a priori* and two series of hypothesized models were defined according to the methodology of Myers et al. (39). First a unidimensional model, representing a global view of the decisional factors (barriers and facilitators) for engaging in PA was tested. Then, a first series of models were examined: (a) a two-factor correlated model (barriers; facilitators); and (b) a bi-factor model with a general factor (decisional balance) and two correlated factors (barriers; facilitators). A second series of models were then tested: (a) a six-factor model correlated with each level of PA barriers and facilitators; and (b) a bi-factor model with a general factor (decisional balance) and six correlated factors (each level of barriers and facilitators). The following indexes and their criteria were reported and evaluated (31): (a) Chi-square value (χ^2^) and Chi-square statistic divided by degrees of freedom (df), with values below 3.00 considered acceptable; (b) root-mean-square error of approximation (RMSEA), for which values close to or below 0.08 were considered acceptable; (c) comparative fit index (CFI) and Tucker-Lewis index (TLI), for which values close to or above 0.90 were considered acceptable; and (d) the Akaike Information Criterion (AIC) and expected cross validation index (ECVI), in which lower values were considered better.

##### 3.1.3.2 Convergent validity

Bivariate Pearson correlations were used. Correlations were interpreted following Cohen's guidelines (40): (a) < 0.10: trivial; (b) 0.10–0.30: small; (c) 0.30–0.50: moderate; (d) 0.50–0.70: large; (e) 0.70–0.90: very large, and (f) >0.90: almost perfect. We computed Pearson correlations for the eight factors of the DBSPA-FSV scale (i.e., global factor of barriers, physical barriers, psychological barriers, socio-environmental barriers, global factor of facilitators, physical facilitators, psychological facilitators, socio-environmental facilitators) and the six types of motivation for PA. Therefore, we expected positive correlations between facilitators and self-determined forms of motivation for PA, and negative correlations between facilitators and non-self-determined forms of motivation for PA. Conversely, we expected negative correlations between barriers and self-determined forms of motivation for PA, and positive correlations between barriers and non-self-determined forms of motivation for engaging in PA.

### 3.2 Results

Skewness and kurtosis were assessed for the *eight* dimensions of the DBSPA-FSV, and the *six* types of motivation for PA. Skewness ranged from −1.69 to 1.61, and kurtosis ranged from – 0.98 to 4.26. Therefore, the normality of data was assumed (33). Subsequent factor analyses employed robust estimators that tolerate moderate departures from normality.

#### 3.2.1 Confirmatory factor analysis

**Figure 1:**
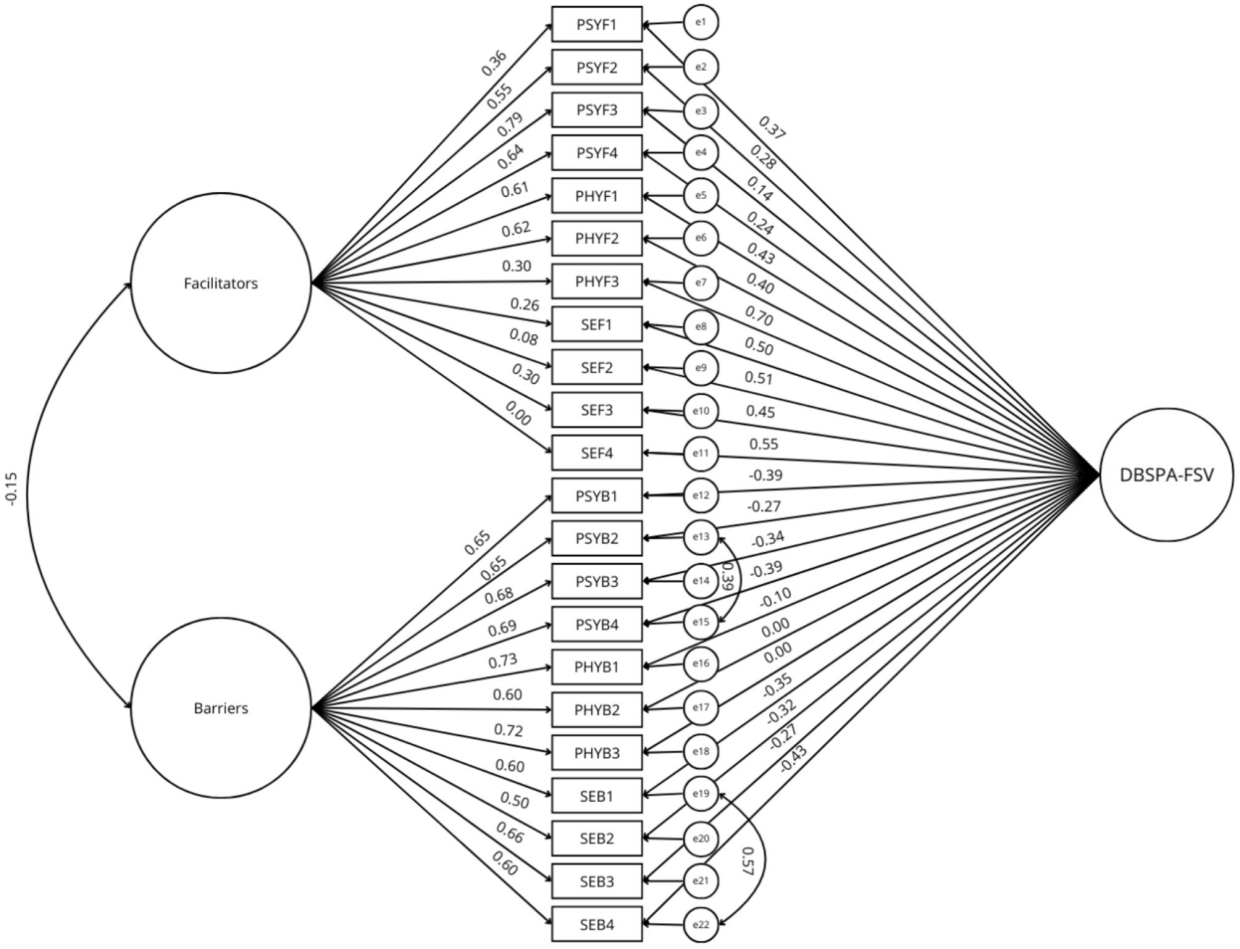
Estimation coefficients and standardized measurement errors of the bi-factor model with one general factor and two correlated factors. PSYF, psychological facilitator; PHYF, physical facilitator; SEF, socio-environmental facilitator; PSYB, psychological barrier; PHYB, physical barrier; SEB, socio-environmental barrier; DBSPA-FSV, decisional balance scale for physical activity in female survivors of violence.

**Figure 2:**
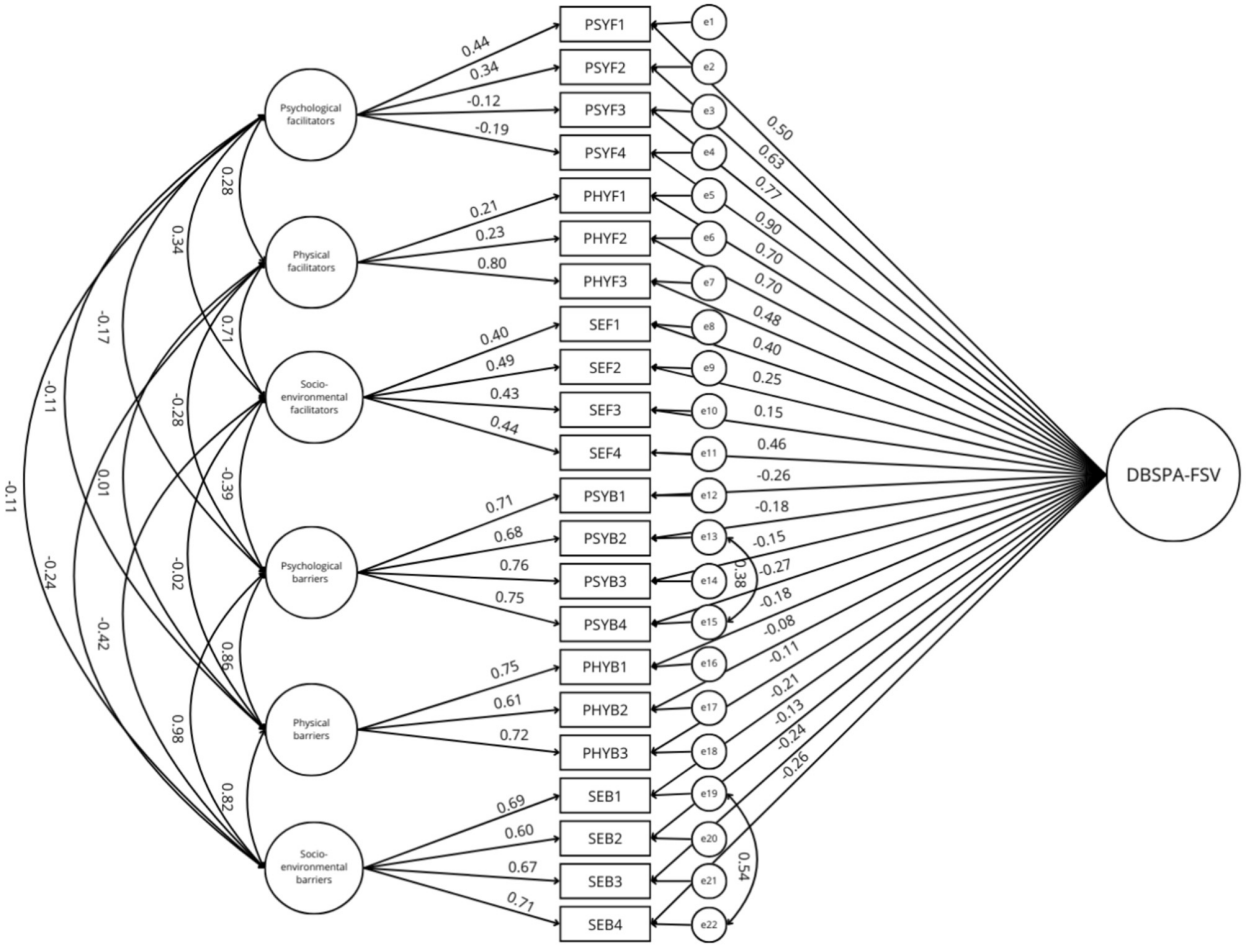
Estimation coefficients and standardized measurement errors of the bi-factor model with one general factor and six correlated factors. PSYF, psychological facilitator; PHYF, physical facilitator; SEF, socio-environmental facilitator; PSYB, psychological barrier; PHYB, physical barrier; SEB, socio-environmental barrier; DBSPA-FSV, decisional balance scale for physical activity in female survivors of violence.

Two series of models were tested with the 22-item version of the DBSPA-FSV scale (*N* = 301). We examined five models: (a) a unidimensional model; (b) a two-factor correlated model; (c) a bi-factor model with a general factor and two correlated factors; (d) a six-factor correlated model; and (e) a bi-factor model with a general factor and six correlated factors. Fit indexes of the tested models are presented in Table 3. The unidimensional model did not present satisfactory fit indexes. The two-factor and the six-factor models presented better fit indexes but still not sufficient to be acceptable. The bi-factor models had the best fit indexes but still did not achieve sufficient fit indexes. Therefore, the modification indexes identified by AMOS were examined, and correlations between the errors of the items PsyB2 and PsyB4, and between the errors of the items SEB1 and SEB4 were added to the models. This addition of correlations between errors, recommended by Byrne (41) was applied to the five models and resulted in acceptable fit indexes for the bi-factor models (see Table 3, and Figures 1, 2). Since the choice of model was based on theoretical reasoning, we chose to keep the two bifactorial models because they presented satisfactory to good indexes, provided the best goodness-of-fit indexes as well as the lowest ECVI and AIC indexes, and they were complementary. This complementarity lies in the fact that various dimensions of the scale can be explored according to the six barriers and facilitators at each level (physical, psychological, and socio-environmental) but also according to the two global dimensions (barriers and facilitators).

**Table 3:** Fit indexes of the different models examined by the confirmatory factor analysis (*N* = 301).

**Models**	***X*^2^ (df)**	**RMSEA**	**CFI**	**TLI**	**AIC**	**ECVI**	**Delta*X*^2^**
Unidimensional	1,432.39 (207)	0.14	0.61	0.57	1,524.39	5.08	
Two-factor correlated	663.85 (206)	0.09	0.86	0.84	757.85	2.53	768.54 (1)^***a^
Six-factor correlated	521.79 (192)	0.08	0.90	0.87	643.79	2.15	910.60 (15)^***a^
Bi-factor (two correlated factors and one general factor)	**434.75** (184)	**0.07**	**0.92**	**0.90**	**572.75**	**1.91**	**229.10 (22)** ^***b^
Bi-factor (six correlated factors and one general factor)	**393.73 (170)**	**0.07**	**0.93**	**0.90**	**559.73**	**1.87**	**128.06 (22)** ^***b^

χ^2^: Chi^2^; DF, degrees of freedom; RMSEA, root mean square error of approximation; 90% CI, confidence interval of RMSEA 90; CFI, comparative fit index; TLI, Tucker-Lewis index of adjustment; AIC, Akaike information criterion; ECVI, expected cross-validation index.

^a^Comparison with the unidimensional model.

^b^Comparison with the X-factor correlated model.

^***^p < 0.001. The selected model is in bold.

#### 3.2.2 Convergent validity

Pearson's correlations between the barriers and facilitators for engaging in PA and the different types of motivation for PA are presented in Table 4. The general and specific dimensions of facilitators were positively correlated with intrinsic, integrated, identified, and introjected types of motivations for PA (*p* < 0.01). They also were negatively correlated with amotivation (*p* < 0.01). Only psychological barriers showed a small significant correlation with external regulation of motivation for PA (*p* < 0.05). The general and specific dimensions of barriers were negatively correlated with intrinsic, integrated, identified, and introjected regulations of motivation for PA (*p* < 0.05), except physical barriers which showed no correlation with identified and introjected regulations of motivation for PA. All barriers were also positively correlated with amotivation and external regulation of motivation (*p* < 0.05).

**Table 4:** Convergent validity results based on Pearson correlations (*N* = 301).

	**Amotivation**	**External regulation of motivation**	**Introjected regulation of motivation**	**Identified regulation of motivation**	**Integrated regulation of motivation**	**Intrinsic motivation**
PSYF	−0.317^**^	−0.140^*^	0.344^**^	0.437^**^	0.386^**^	0.465^**^
SEF	−0.160^**^	−0.058	0.306^**^	0.269^**^	0.420^**^	0.362^**^
PHYF	−0.347^**^	−0.112	0.401^**^	0.491^**^	0.487^**^	0.556^**^
Facilitators	−0.296^**^	−0.064	0.399^**^	0.451^**^	0.456^**^	0.502^**^
PSYB	0.191^**^	0.243^**^	−0.129^*^	−0.150^**^	−0.390^**^	−0.327^**^
SEB	0.173^**^	0.124^*^	−0.197^**^	−0.249^**^	−0.374^**^	−0.348^**^
PHYB	0.148^*^	0.157^**^	−0.068	−0.071	−0.118^*^	−0.176^**^
Barriers	0.193^**^	0.199^**^	−0.150^**^	−0.179^**^	−0.339^**^	−0.324^**^

PSYF, psychological facilitator; PHYF, physical facilitator; SEF, socio-environmental facilitator; PSYB, psychological barrier; PHYB, physical barrier; SEB, socio-environmental barrier; ^**^p < 0.01; ^*^p < 0.05.

### 3.3 Discussion

The confirmatory factor analysis (CFA) showed that the bifactor models with six correlated factors and with two correlated factors both presented the best fit indexes. Thus, these two models are statistically valid. They allow us to examine both an overall decisional balance score, and a simplified two-factor structure (barriers and facilitators) or a more detailed six-factor structure (physical, psychological, and socio-environmental facilitators; physical, psychological, and socio-environmental barriers). These two models provide a comprehensive understanding of the decision balance for each individual. In accordance with the French version of the decisional balance for PA in the general population (27), the DBSPA-FSV can be used to calculate an overall decision balance score based on barrier and facilitator scores but also to identify the scores for each type of barrier and facilitator (physical, psychological, and socio-environmental). Convergent validity was confirmed by relationships between the DBSPA-FSV constructs and the different types of motivations for PA (35) in the expected directions.

## 4 Step 3: reliability of the DBSPA-FSV

The purpose of the third step of the study was to examine the reliability of the 22-item DBSPA-FSV scale. The term “reliability” refers to the consistency or repeatability of measurements (38). The internal consistency of the DBSPA-FSV scale and its temporal stability over a 2-week interval were examined.

### 4.1 Method

#### 4.1.1 Participants and procedure

Participants were 59 female students (M_age_ = 23.00 years, SD = 10.38) of a sport science University of the South of France. Their sociodemographic characteristics are presented in Table 5. Volunteer participants from the main sample (*N* = 301) were asked to complete the 22-item version of the DBSPA-FSV scale on a second occasion within a 2-week interval.

**Table 5:** Characteristics of the participants involved in checking the reliability of the DBSPA-FSV scale (*N* = 59).

**Categories**	***N***	**Mean (SD)**
Age (year)	59	23.00 (10.38)
Height (m)	59	1.65 (0.05)
Weight (kg)	59	61.25 (8.86)
BMI (kg/m^2^)	59	22.27 (2.77)
**Years of education**		
< 12	1	
12	36	
15	11	
≥17	11	
**Exposed to violence**		
No	27	
Yes	32	
Number of years since the violence occurred		4.68 (7.39)

Years of education: < 12: French junior High School Diploma, Professional Aptitude Certificate, Professional Studies Certificate; 12: High School Diploma; 15: Bachelor's degree; ≥17: Master's degree or PhD.

#### 4.1.2 Data analyses

Internal consistency was assessed using Cronbach's alpha coefficients (42) calculated for each dimension of the scale. In line with Aldridge et al. (43), we used the intra-class correlation coefficient (ICC) to assess the test-retest reliability. Values < 0.50 were expected to indicate poor reliability, values between 0.50 and 0.75 moderate reliability, values between 0.75 and 0.90 good reliability, and values >0.90 excellent reliability (40).

### 4.2 Results

Skewness and kurtosis were assessed for the *eight* dimensions of the DBSPA-FSV at the two-time measurements. Skewness ranged from −1.48 to 0.61, and kurtosis ranged from −1.44 to 3.49. Therefore, the normality of data was assumed (33).

#### 4.2.1 Internal consistency

Cronbach's alphas for each subscale were calculated (see Table 6). For both time measurements, Cronbach's alphas ranged from 0.60 to 0.89. All constructs were above 0.70, except socio-environmental facilitators (α_T1_ = 0.66; α_T2_ = 0.62) and physical barriers (α_T1_ = 0.66; α_T2_ = 0.60).

**Table 6:** Intraclass correlation coefficients (*N* = 59).

**Factors**	**Time 1**		**Time 2**		**ICC (95% CI)**
	**M (SD)**	α	**M (SD)**	α	
Physical facilitators	5.08 (0.93)	0.80	4.86 (1.06)	0.84	0.84 (0.72–0.91)
Psychological facilitators	4.94 (0.88)	0.78	4.70 (1.08)	0.84	0.69 (0.48–0.81)
Socio-environmental facilitators	3.99 (1.18)	0.66	4.07 (1.06)	0.62	0.86 (0.76–0.91)
Global facilitators	4.64 (0.84)	0.84	4.52 (0.91)	0.87	0.84 (0.73–0.90)
Physical barriers	2.03 (1.02)	0.66	2.08 (0.99)	0.60	0.73 (0.55–0.84)
Psychological barriers	2.84 (1.37)	0.80	2.91 (1.45)	0.83	0.85 (0.75–0.91)
Socio-environmental barriers	2.67 (1.29)	0.72	2.71 (1.34)	0.74	0.82 (0.70–0.89)
Global barriers	2.56 (1.09)	0.88	2.61 (1.14)	0.89	0.82 (0.70–0.89)

ICC, intraclass correlation coefficients; M (SD), mean (standard deviation); 95%CI, 95% confidence interval; α, Cronbach's alpha.

#### 4.2.2 Test-retest reliability

The ICC results are presented in Table 6. Physical facilitators, socio-environmental facilitators, global facilitators, psychological barriers, socio-environmental barriers, and global barriers presented good reliability (43). Psychological facilitators and physical barriers presented moderate reliability (43).

### 4.3 Discussion

Internal consistency was demonstrated for all subscales (see Table 6), although the socio-environmental facilitators and physical barriers showed only marginally acceptable internal consistency (38). Therefore, the internal consistency was attested. The ICC of the DBSPA-FSV dimensions were moderate to good (44). Therefore, the test-retest reliability was attested.

Internal consistency was demonstrated for all subscales (see Table 6), although the socio-environmental facilitators and physical barriers showed only marginally acceptable internal consistency (38) and therefore should be subject to refinement in future studies. Thus, internal consistency was attested. The ICC of the DBSPA-FSV dimensions ranged from moderate to good (44). Therefore, test-retest reliability was attested.

## 5 General discussion

This study aimed to develop and validate a DBSPA-FSV scale that measures the barriers and facilitators for engaging in PA specific to female survivors of violence. The resulting 22-item scale comprises two subfactors (facilitators and barriers) each encompassing three dimensions: physical, psychological, and socio-environmental. This scale demonstrated satisfactory psychometric properties and represents a key step forward in understanding the psychological factors influencing PA engagement among this population. Existing decisional balance tools are either too generic, failing to capture the specific psychological needs of women exposed to violence—such as the need for safety, trust, and confidentiality—or are too narrowly tailored to other populations (e.g., workplace settings or chronic illnesses), making them unsuitable to this context. Developing a decisional balance scale specifically adapted to women exposed to violence was therefore essential to fill this gap and to support the design of trauma-informed PA interventions.

First, the clarity of all scale items was evaluated to ensure accurate comprehension. Then, an analysis of the dimensional structure was conducted, which led to the selection of two models. The results showed that the bifactor models—with a general factor and either two correlated factors or six correlated factors—presented good fit indices. These validated models offer robust frameworks for understanding both the general and specific dimensions of the barriers and facilitators to engaging in physical activity encountered by women exposed to violence. The validated bifactor models thus provide a comprehensive tool for assessing decisional balance, taking into account both the overall structure and its specific components. As expected, the scale correlated in the anticipated directions with different types of motivation for physical activity (i.e., intrinsic motivation, extrinsic motivation by integrated regulation, identified regulation, introjected regulation, external regulation, and amotivation), demonstrating good convergent validity. These associations support the theoretical coherence of the instrument and open avenues for future research, particularly regarding how different forms of violence may distinctly influence motivational processes related to physical activity. Finally, each of the scale's dimensions showed acceptable internal consistency, as indicated by Cronbach's alpha coefficients. Temporal reliability was also satisfactory across the eight dimensions. Altogether, the validation phases demonstrated the solid psychometric properties of the DBSPA-FSV scale.

This study presents several notable strengths, including the development of an original tool specifically tailored to women exposed to violence, addressing a gap in existing measures of decisional balance for physical activity. The scale is grounded in a solid theoretical framework and was developed through a rigorous, multi-phase psychometric validation process, ensuring both conceptual relevance and methodological robustness. In addition to the strengths of this study, certain limitations must be acknowledged. First, we did not collect data on participants' race or ethnicity, which limits the ability to explore potential interactions between cultural or racial factors and PA engagement. Socioeconomic status was assessed only through the highest educational degree obtained, which provides a partial indicator. Future studies should include more comprehensive measures of socioeconomic and ethnocultural background. Additionally, the type of violence experienced (e.g., psychological, physical, or sexual), the age at which it occurred (e.g., childhood or adulthood), and the duration or frequency of the exposure (e.g., repeated vs. isolated incidents) were not assessed. This lack of contextual factors limits the exploration of potential distinctions. Prior research suggests that such variables can significantly shape psychological outcomes and behavioral responses (45). Future studies should address these questions to allow for a more nuanced understanding of how the nature and timing of violence influence decisional processes related to physical activity. Although this validation highlighted the good psychometric properties of the tool, it is important to emphasize the heterogeneity of behaviors on the continuum of violence experienced by the participants (36). Future studies would be necessary to explore in greater depth differences on the continuum of violence. Temporal stability was attested. However, responses to the barriers and facilitators could evolve over time or vary depending on the severity and recurrence of trauma.

Therefore, a study of their evolution over time would provide valuable insights. Longitudinal studies could assess how interventions targeting specific barriers and facilitators impact PA engagement and overall wellbeing. Furthermore, future research should explore the role of introjected forms of motivation in sustaining long-term PA engagement, given their positive correlations with facilitators despite being extrinsic in nature. Finally, validation of this tool will also allow us to carry out more quantitative studies to examine the psychological correlates of the decisional balance for PA, and to define intervention strategies tailored to survivor profiles.

This scale offers many practical applications. Health and sport professionals can leverage it to design targeted intervention strategies and customize programs to address psychological, physical, and socio-environmental barriers. International validation of the tool in the future could facilitate broader research on PA engagement across diverse cultural contexts, thereby enhancing its overall usefulness. In sum, the DBSPA-FSV scale is a valuable instrument for advancing both theoretical research and applied practices. By contributing to a nuanced understanding of the decisional balance for PA in female survivors of violence, whether through a global index, two broad dimensions (barriers and facilitators), or six specific sub-dimensions, this tool paves the way for the development of innovative strategies to improve their physical and psychological health.

The present research highlights the value of the DBSPA-FSV scale as a novel and psychometrically sound tool to assess the barriers and facilitators for engaging in PA perceived by female survivors of violence. Beyond bridging a critical gap in sport and exercise psychology, this scale has also a broader relevance for public health, trauma-informed care, mental health interventions, and health behavior change research. By capturing the psychological, physical, and socio-environmental factors that influence physical activity engagement in this vulnerable population, the DBSPA-FSV can support the development of targeted, interdisciplinary interventions aimed at promoting physical activity and improving overall wellbeing.

## 6 Conclusion

The DBSPA-FSV scale is a robust and innovative tool for assessing the barriers and facilitators for engaging in PA specific to female survivors of violence. It enriches the sport psychology literature while offering practical value for health and sports professionals. The scale's validation in French-speaking contexts provides a foundation for further international adaptation and application. By providing a deeper understanding of the unique challenges faced by women survivors of violence, the DBSPA-FSV scale could contribute to the development of tailored and impactful interventions for survivors of violence and support them in their journey toward greater PA engagement, improved wellbeing, and better quality of life. Beyond bridging a critical gap in sport and exercise psychology, this scale also has broader relevance for public health, trauma-informed care, mental health interventions, and health behavior change research.

## Data Availability

The raw data supporting the conclusions of this article will be made available by the authors, without undue reservation.
